# Intradermal injection of tranexamic acid versus platelet-rich plasma in the treatment of melasma: a split-face comparative study

**DOI:** 10.1007/s00403-023-02580-y

**Published:** 2023-03-01

**Authors:** Israa Gomaa Abd Elraouf, Zakaria Mahran Obaid, Ibrahim Fouda

**Affiliations:** 1 Dermatology, Venereology and Andrology Department, Itay Elbaroud General Hospital, Albehira, Egypt; 2 Dermatology, Venereology and Andrology Department, Damietta Faculty of Medicine, Al-Azhar University, Damietta, Egypt

**Keywords:** Melasma, mMASI, PRP, Tranexamic

## Abstract

Millions of people throughout the world suffer from the acquired condition of hyperpigmentation known as melasma. Melasma is characterized by symmetrically oriented hyperpigmented macules and patches. Many treatment options are available with variable degrees of efficacy and tolerability. The aim of the work was to evaluate and compare the effectiveness and safety of intradermal tranexamic acid (TXA) versus intradermal platelet-rich plasma (PRP) in the treatment of various types of melasma. The current split-face prospective study included 40 cases with melasma. Tranexamic acid (TXA) was injected intradermally into the right side of the face by using a concentration of 4 mg/ml, while platelet-rich plasma (PRP) was injected intradermally into the left side. In both sides, a total of three sessions of treatment were provided, once every 4 weeks. Digital photographs were taken before each treatment session and 3 months after the last session. The modified melasma area severity index (mMASI) grading system and dermoscopy were used to assess the improvement in the condition. The disease severity and percentage of improvement were assessed by mMASI score before and after therapy across both sides of the face. along with determining the degree of satisfaction and side effects among the included cases. The mean mMASI score before therapy in the TXA side was 4.59 ± 2.87, while in the PRP side, the mean mMASI score before therapy was 4.72 ± 2.72 with no statistically significant difference between the two sides (*p* = 0.841). After 3 months of treatment, the mean mMASI score in the TXA-treated side was 2.49 ± 1.58 with a mean percentage of decrease of 45.67 ± 8.10%, while in the PRP side, the mean mMASI score after treatment was 2.17 ± 1.41 with a mean percentage of decrease of 53.66 ± 11.27%. There was a high statistically significant decrease in the mMASI score after treatment on both sides (*p* < 0.001); however, the percentage of score reduction in the PRP side compared to the TXA side was statistically higher. Intradermal injection with PRP revealed higher efficacy in the treatment of melasma as compared to TXA injection with no significant difference regarding the associated side effects.

## Introduction

Melasma is a common acquired disorder characterized by hyperpigmented macules or patches that are most frequently found on the mandibular, malar, and centrofacial regions—forehead, nose, upper lip, and chin [1].

Melasma pathogenesis is complex and poorly understood. Various underlying risk factors for developing melasma have been described, including oral contraceptives, sun exposure, hormonal changes during pregnancy, and genetic predisposition. Moreover, to transfer melanosomes to keratinocytes through the tyrosinase enzyme, which controls the production of melanin, the pathogenesis of melasma could be caused by melanogenesis dysfunction, either through increased exposure to melanogenic factors or through increased sensitivity to risk factors [2, 3].

Managing melasma started by prevention using sunscreens. Therapeutic approaches of melasma utilizing chemical peel and topical drugs such as hydroquinone, azelaic acid, retinoids, corticosteroids and arbutin either alone or in combinations have been commonly employed as the main lines of treatment [4].

Tranexamic acid is a synthetic lysine derivative that inhibits plasminogen activation and has anti-fibrinolytic effects by blocking lysine binding sites on plasminogen molecules, which prevents plasminogen from interacting with formed plasmin and fibrin and stabilizes the preformed fibrin meshwork created by secondary hemostasis. It acts on melasma by preventing keratinocytes' plasmin activity after exposure to UV light. Therefore, inhibiting plasminogen's ability to bind to keratinocytes will reduce the amount of free arachidonic acid, which is essential for the formation of prostaglandins and improve tyrosinase activity [5, 6]^.^

Platelet-rich plasma (PRP) is described as a tiny volume of autologous plasma with a high concentration of platelets, obtained by centrifuging autologous blood and then suspending the platelets to release platelet-derived growth factor, which increases skin volume as a result of angiogenesis and collagen synthesis and also improves melasma. Transforming growth factor beta (TGF-β1) released from α-granules in platelets has been shown to cause significant inhibition of melanin synthesis through delayed extracellular signal-regulated kinase activation [7–9].

Many studies have been conducted to evaluate and compare platelet-rich plasma and tranexamic acid. This study aimed to evaluate the efficacy of intralesional PRP versus TXA in the treatment of melasma through dermoscopic and clinical evaluation by using the mMASI score.

## Patients and methods

This is a split-face prospective study that was conducted on 65 clinically diagnosed patients with melasma randomly selected from an outpatient clinic; 25 cases were excluded from the study by exclusion criteria and 40 cases received treatment and follow-up with no dropout.

The study included 40 cases with Fitzpatrick skin types III and IV, from both genders with all types and degrees of facial melasma. Tranexamic acid intradermal injections were administered to the right side of the face, whereas PRP intradermal injections were administered to the left side.

Pregnant and breastfeeding females, those who were taking contraceptive pills, those of any known bleeding problems or concurrent anticoagulant usage at the time of the research, patients who had used any form of melasma treatment, whether oral or topical, within the previous 3 months, patients with established platelet dysfunction syndrome, patients with critical thrombocytopenia (< 50,000/ul), and patients on any hormonal therapy (as in the case of hormone replacement therapy for menopausal females and treatment of endometriosis) were excluded from the study.

All patients underwent a full history-taking including personal history (age, marital status, occupation, and family history), history of the lesion (duration of the lesion, predisposing factors), and dermatological examination to assess the type and severity of the melasma.

### Assessment of disease severity

Digital photographs were taken for the lesions before and after the end of treatment by using Apple iPhone 11 Pro Max- camera 12 MP.

Wood’s lamp (Lumio^®^UV 3Gen- dermlite) and dermoscopic (by DermLite DL4 dermoscope) examinations were conducted on all patients before the treatment to determine the type of melasma (epidermal, dermal, and mixed) as well as the vascular and pigmentation components of melasma. The disease severity was assessed using the modified melasma area and severity index (mMASI).

The study was conducted in accordance with Helsinki standards as revised in 2013. The study was conducted after obtaining the approval from the Ethics Committee of Damietta Faculty of Medicine IRB (00,012,367), Al-Azhar University, Egypt, and after obtaining an oral informed consent from the included cases.

## Treatment regimen

### Method of preparation and TXA injection

The right side of the face received intradermal injection with tranexamic acid using 100U/ml insulin syringe, Tranexamic acid was collected from Kapron^®^ Ampoules, Amoun Pharmaceutical Company, with a concentration of 100 mg/ml. The concentration of 4 mg/ml of tranexamic acid was obtained by drawing about 4 mg of the drug into a 100U/ml insulin syringe and diluting it with saline to a volume of 1 ml. A topical anesthetic cream (Emla 5% cream, AstraZeneca Pharmaceutical Company) was applied to the face and left for 30 min. About 0.05 ml was injected intradermally into the lesions at 1 cm interval to a maximum 8 mg to the entire affected area.

### Method of preparation and PRP injection

The left side of the face received intradermal injection of PRP: 10 mL of venous blood was drawn from the antecubital vein and placed in tubes containing sodium citrate 3.2% as an anticoagulant (sodium citrate 9NC, VACO MED) under completely aseptic conditions before being double spun. The second centrifugation was quicker at 4000 rpm for 5 min after the first one which was slower at 3000 rpm for 7 min. A concentration of activated PRP was then obtained by aspirating the resulting plasma and activating it with calcium chloride (CaCl2) in a ratio of 0.1 mL of CaCl2 to 0.9 mL of PRP. A 30-gauge needle was used for the injection, with a session limit of 1 mL.

## Clinical assessment and follow-up

- The procedure was repeated three times at monthly intervals (0, 1, and 2 months), and then the patients were followed up every month for another 3 months to detect any recurrence.
- Patients were counseled to limit their exposure to the sun and use a broad-spectrum sunscreen with a sun protection factor above 30 during daytime throughout the entire treatment period.
- Assessment was done by two blinded investigators.

## Statistical analysis of data

The data collected were coded, processed, and analyzed with SPSS version 27 for Windows^®^ (Statistical Package for Social Sciences) (IBM, SPSS Inc., Chicago, IL, USA). Qualitative data as number (frequency) and percent were presented. The Chi-square test (or Monte Carlo test) made the comparison between groups.

The Kolmogorov–Smirnov test tested quantitative data for normality. To compare two independent groups with parametric quantitative variables, independent samples *t* test was used and Mann–Whitney *U* test was used if the data were non-parametric. To compare two dependent groups with parametric quantitative variables, paired samples *t* test was used and Wilcoxon-signed rank test was used if the data were non-parametric. For all tests, *P* values < 0.05 are considered significant.

## Results

As shown in Table 1, there were 39 females among the included cases (97.5%) and 1 male (2.5%). The mean age of the cases was 39.20 ± 5.22 years with range between 28 and 52 years. The highest percentage of the cases showed a gradual onset (97.5%) and a progressive course (70%). The mean duration of the disease among the included cases was 4.54 ± 3.02 years with range between 8 months and 12 years.

**Table 1:** Baseline characteristics of the studied patients (*n* = 40)

	No	%
Sex		
Male	1	2.5
Female	39	97.5
Age (years)		
Min.–Max	28.0–52.0	
Mean ± SD	39.20 ± 5.22	
Median (IQR)	38.50 (36.0–41.50)	
Onset		
Gradual	39	97.5
Sudden	1	2.5
Course		
Progressive	28	70.0
Stationary	12	30.0
Duration (years)		
Min.–Max	0.67–12.0	
Mean ± SD	4.54–3.02	
Median (IQR)	4.50 (2.0–6.50)	
Site		
Malar	39	97.5
Mustache	24	60.0
Frontal	22	55.0
Risk factor		
Negative	15	37.5
Sun exposure	15	37.5
Hormonal	10	25.0
Family history	14	35.0
Associated skin disorders	1	2.5
Systemic disease	1	2.5

Regarding the site, the malar region was affected in 39 cases (97.5%), mustache in 24 cases (60%), and then the frontal area in 22 cases (55%). Sun exposure was the most common risk factor in 37.5% of the cases, followed by hormonal causes in 25% of the cases. Positive family history was reported in 35% of the cases. One case showed associated skin disease (adult hormonal acne) and also one case showed other associated chronic diseases (diabetes mellitus).

As shown in Table 2, the median (IQR) of mMASI score before treatment in the TXA group was 4.20 (2.18–6.60), while in the PRP group, the median (IQR) of mMASI score before treatment was 3.59 (2.73–7.085) with no statistically significant difference between the two groups (*p* = 0.841). After treatment, the median (IQR) of mMASI score in the TXA-treated group was 2.10 (1.30–3.38) with mean percentage of decrease of 45.67 ± 8.10%, while in the PRP group, the median (IQR) mMASI score after treatment was 1.725 (1.025–3.15) with mean percentage of decrease of 53.66 ± 11.27%,.

**Table 2:** Comparison between TXA and PRP according to mMASI score before and after treatment

	TXA	PRP	*P*
mMASI score before treatment			
Median (IQR)	4.20 (2.18–6.60)	3.59 (2.73–7.085)	0.841#
mMASI score after treatment			
Median (IQR)	2.10 (1.30–3.38)	1.725 (1.025 – 3.15)	0.350#
P1 (comparison to baseline value in each group)^a^	< 0.001	< 0.001	
Percent of score reduction (%)	45.67 ± 8.10	53.66 ± 11.27	0.005* ^

#Mann–Whitney *U* test

^Independent samples (Student’s *t* test)

^a^Wilcoxon signed-rank test

*Statistically significant (*p* < 0.05)

**Fig. 1:**
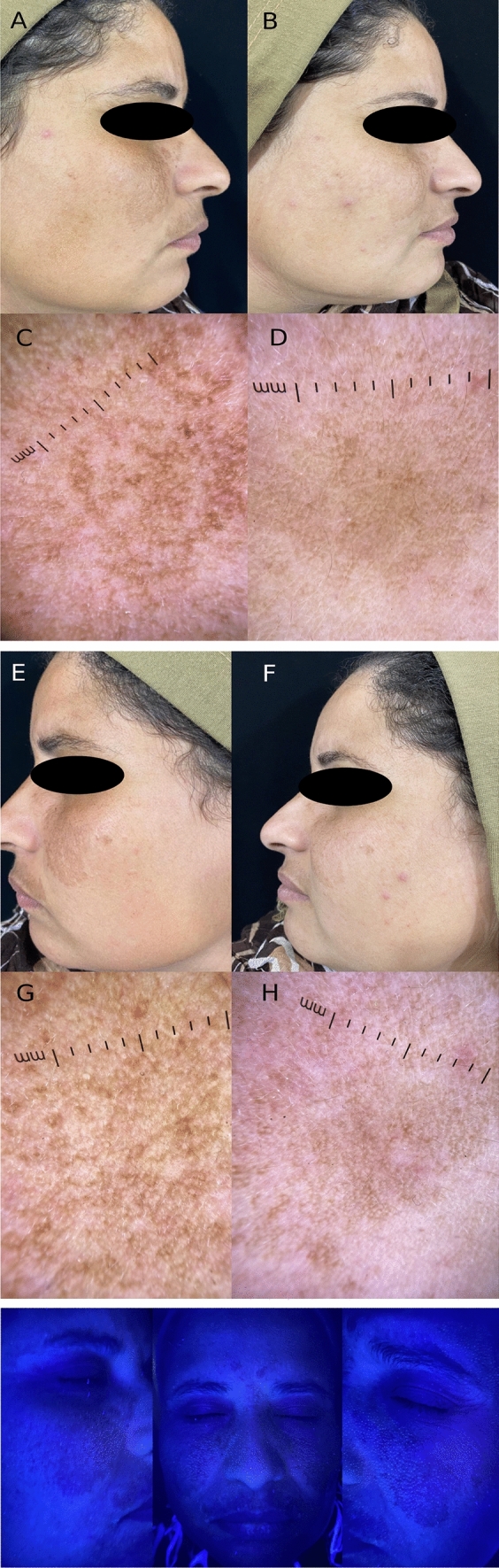
A 40 years old female skin type IV, suffered from mixed melisma, **A** the right side of the face before treatment with mMASI score = 4.8. **B** Right side after treatment with tranexamic acid intradermal injection in mMASI score = 1.5, **C** Right side dermoscopic features before treatment showing scattered island of dark brown pseudo-reticulate pattern, with sparing of the follicular region. **D** Right side dermoscopy after treatment showing decrease in intensity of pigmentation darkness to light brown. **E** The left side of the face before treatment with mMASI score = 5.1. **F** The left side after treatment with PRP intradermal injection showing marked improvement and decrease in mMASI score = 1.2, **G** The left side dermoscopic features showing scattered island of dark brown pseudo-reticulate pattern, with sparing of the perifollicular region and telangectasia. **H** The left side after treatment showing decrease in the intensity of pigmentation darkness and telangectasia. Wood’s lamp examination showing accentuation of pigmentation (Mixed melasma)

**Fig. 2:**
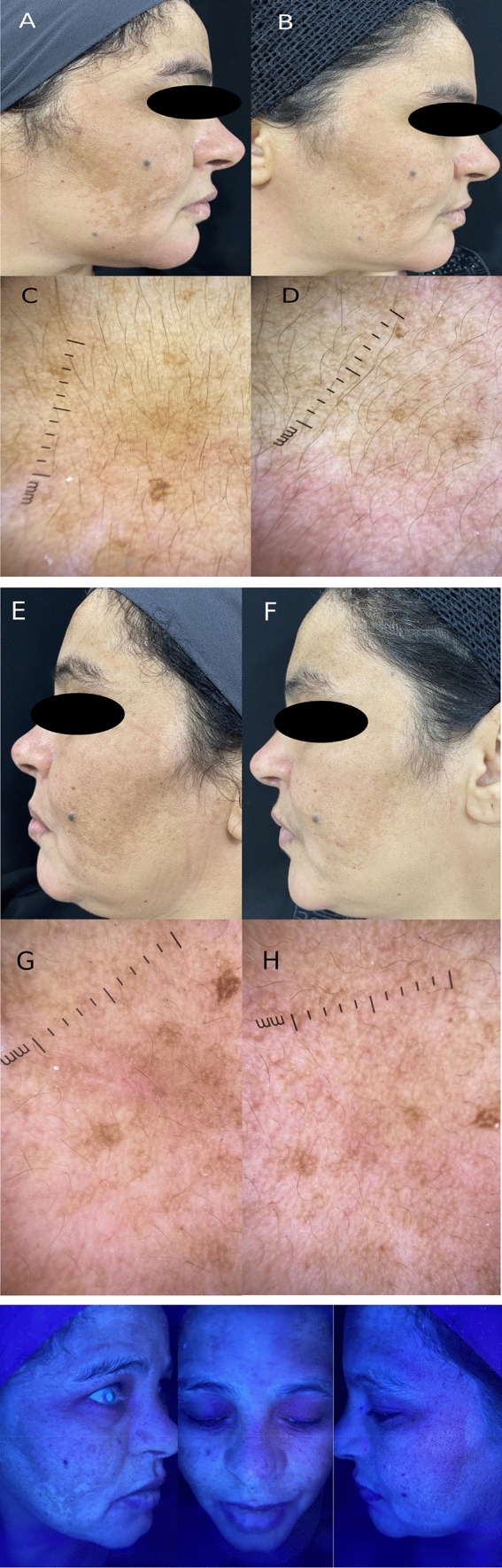
A 48 years old female skin type III, suffered from mixed melasma: **A** the right side of the face before treatment with mMASI score = 6.1. **B** Right side after treatment with tranexamic acid intradermal injection showing mild improvement and decrease in mMASI score = 3.1, **C** Right side dermoscopic features before treatment showing both epidermal features in the form of reticulate and pseudo-reticulate pattern and dermal features in the form of light brown patches and archiform structures. **D** Right side dermoscopy after treatment showing decrease in the intensity of pigmentation darkness. **E** The left side of the face before treatment with mMASI score = 4.6. **F** The left side after treatment with PRP intradermal injection showing marked improvement and decrease in mMASI score = 1.6. **G** The left side dermoscopic features showing both epidermal features in the form of reticulate and pseudo-reticulate pattern and dermal features in the form of light brown patches and archiform structures. **H** The left side after treatment showing marked decrease in the intensity of pigmentation darkness from dark to light brown. Wood’s lamp examination showing minimal accentuation of pigmentation (Mixed melasma)

There was high statistically significant decrease in the mMASI score in each of the two sides (*p* < 0.001), but the percentage of score reduction in the PRP side compared to the TXA side was statistically higher (Fig. 1and 2).

Regarding the degree of satisfaction (Table 3), in the TXA side, poor satisfaction was reported in 5% of the cases, slight satisfaction in 37.5%, and satisfaction in 57.5%, while in the PRP side, poor satisfaction was reported in 17.5% of the cases, slight satisfaction in 45%, and satisfaction in 37.5% with no statistically significant difference.

**Table 3:** Comparison between the T.X.A and P.R.P according to side effects and satisfaction

	T.X.A		P.R.P		*P* value
	No	%	No	%	
Satisfaction					
Poorly satisfied	2	5.0	7	17.5	0.097 ^@^
Slightly satisfied	15	37.5	18	45.0	
Satisfied	23	57.5	15	37.5	
Side effects					
No	10	25.0	25	62.5	0.001^*^^¥^
Pain	25	62.5	3	7.5	< 0.001^*^^©^
Erythema	22	55.0	13	32.5	0.043^*^^¥^

^@^Monte-carlo test

^¥^Chi-square test

^©^Fischer’s exact test

*Statistically significant (*p* < 0.05)

The reported side effects in the TXA side included pain in 62.5% and erythema in 55%, while in the PRP side, pain was reported in 7.5% and erythema in 32.5%. No statistically significant difference was found with regard to the side effects**.**

Regarding dermoscopic assessment on both treated sides, the dermoscopic characteristics showed a significant decrease of granular pigmentation, pseudo-network pigmentation, arcuate or annular perifollicular pigmentation, and light brown reticular networks, erythema, and telangectasia, which became less evident with treatment (Fig. 1b and 2b).

## Discussion

Melasma is regarded as one of the more difficult to treat diseases despite having a variety of treatment options, none of which are regrettably totally effective.

This split-face study included 40 cases with melasma, in which the right side of the face received intradermal tranexamic acid injection, while the left side received intradermal PRP injection.

In France, Pistor invented the mesotherapy technique, which is now widely used in medicine [10]. It is a minimally invasive method of drug delivery that consists of multiple intradermal or subcutaneous injections of a mixture of compounds “mélange” in minute doses. Plant extracts, homeopathic agents, pharmaceuticals, vitamins, and other bioactive substances can be used, but alcohol- or oil-based substances should not be used for mesotherapy because of the risk of cutaneous necrosis. [11].

In 2006, Lee et al. published the first study demonstrating the viability of localized microinjections of TXA for melasma. While in 2014, Cayrili and colleagues published the first description of the advantageous benefits of PRP as a standalone therapy for melasma. [12, 13]

In the current study, there were 39 females among the included cases who represented 97.5% of the cases.

This was in agreement with *Fawzy Ewaiss *et al.’s study, which showed that all the included cases in their study were females [14], and the studies by *Serra *et al*. (2018) *[15]and *Jin *et al*. (2019)* [16]*.*

In this study, the mean mMASI score after treatment did not reveal a difference between the two sides that is statistically significant (2.49 ± 1.58 and 2.17 ± 1.41 in the TXA side and PRP side, respectively). However, the percentage of score reduction was higher in the PRP side (53.66 ± 11.27) as compared with the TXA side (45.67 ± 8.10) (*p* = 0.014).

Our results agreed with those of *Mumtaz *et al*.*, who showed that Intradermal PRP was significantly better than intradermal tranexamic acid in the management of melisma. The mean mMASI score at baseline was 29.84 ± 5.14 vs. 29.56 ± 4.39 in the intradermal platelet-rich plasma (PRP) group and tranexamic acid group, respectively, with no statistically significant difference between the two groups (*p* = 0.21). mMASI was significantly better in the PRP group at 4 weeks in which *p* = 0.01. Mean mMASI was 12.81 ± 1.78 vs. 18.38 ± 3.50, *p* = 00,001 at 12 weeks and 8.72 ± 3.40 vs. 14.97 ± 4.33, *p* = 0.02 at 24 weeks in the PRP group and tranexamic acid group, respectively [17].

Our results were in line with those of *Gharieb *et al*.*, who showed that there was a statistically significant difference, as evidenced by the mean difference in mMASI scores between the two groups (*p* = 0.017). Patients who were treated with PRP saw more improvement. As a result, microneedling with PRP has a more potent effect than microneedling with TXA [18].

In the current study, the mean mMASI score decreased from 4.59 ± 2.87 before treatment to 2.49 ± 1.58 after treatment in the TXA with high statistically significant difference (*p* < 0.001).

Similar results were reported by *Badran *et al*.* who used two different concentrations of TXA (4 mg/mL and 10 mg/mL). Their findings indicated that the intradermal injection of TA (in both concentrations) leads to significant improvement of melisma, and a higher concentration of TXA injection results in better improvement, but the difference is non-significant [19].

In this study, the mean mMASI score decreased from 4.72 ± 2.72 before treatment to 2.17 ± 1.41 after treatment in the PRP with high statistically significant difference (*p* < 0.001).

This was in accordance with the results of *Hofny *et al*.*, who reported that the use of PRP is linked to a considerable to outstanding improvement in melasma patients, as demonstrated by the significant decline in the baseline MASI and mMASI scores, and in accordance with the levels of patients' satisfaction. Only two patients (8.7%) were unsatisfied with their improvement, whereas 39.1% of patients were very satisfied, 39.1% were satisfied, 13.1% were slightly satisfied, and 39.1% were satisfied overall [20].

In the study by *Gamea *et al*.,* who compared the efficacy of topical tranexamic acid 5% in liposome base alone versus its combination with intradermal platelet-rich plasma (PRP) for melasma treatment, patients of the combined TXA + PRP group were more satisfied with the treatment outcome than those of the TXA group and the difference was statistically significant [21].

In a study by Zhang et al., who investigated the effect of platelet-rich plasma (PRP) combined with tranexamic acid (TXA) in the treatment of melasma and its effect on the serum levels of vascular endothelial growth factor (VEGF), endothelin-1 (ET-1), and melanin-stimulating hormone (MSH), they reported that PRP combined with TXA can improve the treatment outcome, maintaining normal levels of VEGF, ET-1 and MSH, and reducing the recurrence rate [22].

Our study was concordant with the study of Patil and Bubna, who investigated the intradermal injection of TXA and PRP in the treatment of melisma in two groups, with 18 in the TXA group and 15 in the PRP group. On comparing the mean reduction for each therapy in both scoring systems (MASI and mMASI), it was observed to be slightly greater for the PRP treatment arm. However, *p* values were not statistically significant (mean mMASI: *p* = 0.3, mean mMASI: *p* = 0.4) [23].

Polat and Sarac studied 60 melasma patients. 30 were treated with oral TA and 30 were treated by intradermal injection of PRP for 3 months. A statistically significant improvement was found in the mMASI score consistent with the literature and it was observed that the mMASI score decreased by 65.7% in the TXA group and 54.6% in the PRP group [24].

Due of its autologous nature, PRP therapy has a higher safety profile. A further benefit is that the abundance of growth factors which facilitate a number of mechanisms that result in facial rejuvenation.

In this study, the reported side effects in the TXA group included pain in 62.5% and erythema in 55%, while in the PRP group pain was reported in 7.5% and erythema in 32.5%, with no statistically significant difference between the two groups.

Unlike the previous studies, the advantages of this study are that it is a split-face comparative study, so each subject acts as his or her own control. This can minimize the risk of confounding because all interventions were measured on the same participants and a smaller number of patients were required in comparison to parallel-group studies. Furthermore, intradermal injections of TXA (rather than oral or topical administration) and dermoscopy-based evaluation of the severity and improvement of melasma were the contrasting features observed in our study.

## Conclusion

Due to the fact that melasma is a localized condition of hyperpigmentation, we believe intradermal TXA injections should be considered over an oral route.

Based on our findings, it could be concluded that intradermal injection of both TXA and PRP was associated with high statistically significant decrease in the disease severity. The efficacy of PRP in decreasing the disease severity was superior to TXA with no statistically significant difference with regard to the associated side effects.

Therefore, if there are no contraindications to PRP administration, we believe PRP could be a good alternative for treating melasma.

### Limitations of the study

- Absence of long follow-up periods.
- Evaluation of mMASI score between the first and last session.
- Great number of treatment sessions.

## Data Availability

The datasets generated during and/or analysed during the current study are available from the corresponding author on reasonable request.
